# Perceived eating norms and vegetable consumption in children

**DOI:** 10.1186/s12966-015-0296-z

**Published:** 2015-10-14

**Authors:** Maxine Sharps, Eric Robinson

**Affiliations:** Psychological Sciences, University of Liverpool, Eleanor Rathbone Building, Liverpool, L69 7ZA UK

**Keywords:** Social eating, Perceived eating norms, Social norms, Food intake

## Abstract

**Background:**

Beliefs about the eating behaviour of others (perceived eating norms) have been shown to influence eating behaviour in adults, but no research has examined whether young children are motivated by perceived eating norms.

**Findings:**

Here we investigated the effect on vegetable intake of exposing children to information about the vegetable intake of other children. One hundred and forty three children aged 6–11 years old took part in a between-subjects experiment. Children were exposed to information suggesting that other children had eaten a large amount of carrots, no carrots, or control information. Children ate more carrots when they believed that other children had eaten a large amount of carrots, compared to all other conditions.

**Conclusions:**

Perceived eating norms can influence vegetable intake in young children and making use of eating norms to promote healthier eating in children warrants investigation.

**Electronic supplementary material:**

The online version of this article (doi:10.1186/s12966-015-0296-z) contains supplementary material, which is available to authorized users.

## Findings

### Introduction

Eating behaviours are believed to develop through social learning during childhood, where the presence of dining companions, including parents, peers and siblings, influences the development of food preferences and eating behaviours [1–3]. In adulthood, the eating behaviour of other people is thought to be a strong influence on what and how much we eat [4]. Research consistently supports that people are strongly influenced by the eating behaviour of peers, with adults, adolescents, and children found to adjust their intake to that of a present peer [4–7].

Beliefs about the eating behaviour of others, known as perceived eating norms, have been found to influence eating behaviour in adults [8–10]. For example, Robinson, Fleming, and Higgs (2014) exposed adults to information about eating norms through social norm-based messages suggesting the healthier eating behaviour of peers. Adults ate more fruit and vegetables when they were led to believe that their peers had eaten a large amount of fruit and vegetables [9].

Although research suggests that children adjust their own intake of high calorie snack foods to that of a present peer [7, 11], no research has examined whether children are motivated by perceived eating norms. Since vegetable consumption is often low in young children [12, 13] and dietary habits developed during childhood track into adolescence and adulthood [14, 15] investigating novel ways to increase vegetable intake is of importance.

In the present study we examined whether perceived eating norms influence vegetable intake in children. Children were exposed to information concerning the amount of carrots that other children had eaten. In line with previous studies in adults, we hypothesised that children may be motivated to eat in line with perceived eating norms and increase their vegetable consumption when they believed that other children had been eating a large amount of vegetables.

## Method

### Participants

143 children (51 % females) aged 6–11 years old (9.03 years, SD ± 1.28) were recruited from two Primary schools in North-West England. The sample consisted of 128 normal-weight and 15 overweight children. Children were led to believe that the study was investigating their game-playing abilities. The study was approved by the University of Liverpool Research Ethics Committee. Fully-informed consent was provided.

### Experimental design

Children were randomised (using an online research randomiser; www.randomizer.org) into one of four conditions (high intake norm, low intake norm, no norm, control) in a between-subjects design. The study adopted a remote-confederate design [8, 10], where children were exposed to a fictitious participant information sheet containing information about six previous children (participant number, date of birth and gender). In the high intake, low intake and no-norm conditions the information sheet included the column ‘Carrots (amount eaten)’. In the high intake norm condition this stated ‘all’, in the low intake norm condition this stated ‘none’, and remained empty in the no-norm condition. The column was present in the no-norm condition to rule out that information suggesting that other children’s intake had been monitored would affect intake, but was not present in the final condition; control condition. All children were also exposed to a bowl. In the high intake and low intake norm conditions the bowl contained a single remaining carrot, or was full, to corroborate with the amount of carrots other children had supposedly been eating. In the no-norm condition and the control condition the bowl contained an item unrelated to food (pens).

### Questionnaire measures

#### Fruit and vegetable consumption and liking

To assess usual fruit and vegetable intake, the Day in the Life questionnaire (DILQ) was administered, which is a validated and reliable twenty-four hour recall measure for use in children [16]. The DILQ is a supervised exercise which uses words and pictures to encourage the child to recall and describe a range of activities, including their entire food intake, for the previous day [16]. We also included measures for the children’s liking of fruit and vegetables (e.g., how much do you like fruit/ vegetables/ carrots?) with 5 response options ranging from ‘not at all’ to ‘a lot’, and questions about their beliefs about the fruit and vegetable consumption of other children (e.g., how many fruit and vegetables do you think other children eat every day?) with response options ‘none’, ‘1’, ‘2–3’, ‘4’, ‘5 or more’. These questions were assessed using smiley-face Likert-style scales, and were based on questions used by Lally et al. [17].

#### Manipulation check

To examine whether the norm manipulation was successful, i.e., it caused children to believe that other children had either eaten a large amount of carrots or no carrots, children were asked ‘how many carrots do you think other children ate in the study’ and were presented with three choices ‘almost all’, ‘some’ and ‘none’, alongside a photograph of either an empty, half full or full bowl of carrots.

#### Procedure

Children were tested individually during weekdays between 9 am and 3.30 pm at a primary school. Children were informed that the researcher was interested in how well they played a game. First, the researcher presented the child with the fictitious participant information sheet, and completed the date of birth and gender columns with the child. In the norm conditions the researcher pointed out the ‘Carrots (amount eaten)’ column and explained that this now did not need to be completed, and had only been completed previously for carrot buying purposes. The researcher then pointed out the intake of previous children in the high and low intake norm conditions. In all conditions the researcher ‘noticed’ the bowl on the table, and in the high and low intake norm conditions, the researcher described the intake of previous participants to the child. Next, the child was presented with a bowl of carrots and was informed that they could have as many or as few of the carrots as they wished while the researcher arranged the game. The child was left alone for 7 min to consume as many carrots as they wished. After the 7 min, the researcher explained that the game involved trying to find pairs of animal images. The carrots were removed from the table and the child was left to play the game for three minutes. On return, the researcher congratulated the child on their performance in the game, to corroborate with the cover story. Finally, the researcher asked the child what they thought the aims of the study were, completed the questionnaire measures with the child, and measured the child’s height and weight. (BMI was calculated as weight (kg)/height (m^2^). Using internationally recognised criteria for children [18] healthy-weight, overweight and obesity were defined based on age and sex-specific BMI cut-off points equivalent to adult BMI of 25–30 kg/m^2^ respectively). The bowls of carrots were weighed pre and post-consumption to determine the amount of carrots eaten (in grams).

## Results

No differences (*p*s > .05) were found between the conditions for BMI, age or gender. See Table 1. Children’s mean fruit and vegetable consumption was 1.58 (SD ± 1.53) pieces per day, and children believed that other children ate 2–3 pieces of fruit and vegetables a day on average (2.63, SD ± .68). In addition, children tended to report that they liked carrots (4.13, SD ± 1.24 on the 1–5 scale).

**Table 1:** Mean values (SDs) and statistical test results for BMI, age, gender, and manipulation check

Variables	High intake norm	Low intake norm	No norm	Control	Test statistic and *p*-value
	(*n* = 36)	(*n* = 37)	(*n* = 35)	(*n* = 35)	
BMI (*z*-score)	.30	.35	.42	.10	F(3,139) = .77, *p =* .51
	(1.10)	(.81)	(.96)	(.87)	
Age (years)	9.07	9.20	9.01	8.85	F(3,139) = .45, *p =* .72
	(1.35)	(1.14)	(1.21)	(1.50)	
Gender					
Boys (n)	17	20	18	15	*X* ^2^(3, *n* = 143) = 1.04, *p* = .79
Girls (n)	19	17	17	20	
Belief about amount of carrots eaten by other children (manipulation check) ^a^	2.7	1.6	2.0	2.0	F(3,139) = 45.1, *p* < .001
	(.48)	(.55)	(.24)	(.24)	

^a^Children selected one of three options regarding their beliefs about the amount of carrots eaten by other children: almost all, some, or none. A higher mean corresponds to a belief that other children had eaten a large amount of carrots

### Manipulation checks

No children guessed, or came close to guessing the aims of the study. To check whether children believed the norm manipulation, a one-way ANOVA was conducted on children’s beliefs about the amount of carrots eaten by other children. There was a significant main effect of condition [F(3,139) = 45.11, *p* < .001, ƞp^2^ = .49]. Children in the high intake norm condition believed that other children had eaten significantly more carrots than children in the other three conditions (*p* < .001). Children in the low intake norm condition believed that other children ate significantly less carrots than children in the remaining two conditions (*p* < .001). See Table 1.

### Carrot intake

Using One-Way ANOVA there was a significant main effect of condition on the amount of carrots eaten (in grams) [F(3,139) = 6.90, *p* < .001, ƞp^2^ = .13]. Children in the high intake norm condition ate significantly more carrots than children in all other conditions (*ps* < .01). There were no other significant between-condition differences. See Table 2 for mean intake figures.

**Table 2:** Mean (SDs) intake of carrots (grams)

Condition	Mean intake (grams)
High intake norm (*n* =36)	57.72 (39.02)
Low intake norm (*n* = 37)	27.14 (32.40)*
No norm (*n* = 35)	31.50 (27.36)*
Control (*n* = 35)	29.00 (31.80)*

*indicates significant difference at *p* < .01 to high intake norm condition

### Other variables

We also examined whether BMI, child age, gender, liking of carrots, or usual fruit and vegetable intake moderated the effect of condition. We found no evidence that any of the other variables interacted with condition to predict carrot intake (*p*s > .05), suggesting that the effect of perceived carrot intake was observed consistently across most children. See Additional file 1.

## Discussion

In the current study children were influenced by perceived eating norms regarding their peers’ vegetable intake; when children believed that others had been eating a large amount of carrots they ate more than children in the other conditions. These results suggest that seeing information which suggests that other children have eaten a large amount of vegetables, influences children to increase their own vegetable intake.

Although previous work has suggested that children may mimic the eating behaviour of a present peer [7], this study provides the first evidence that children are influenced by perceived eating norms regarding the vegetable intake of their peers. These results are consistent with previous studies in adults, whereby, exposing adults to information suggesting that previous adults had eaten a large amount of food, influenced food intake [4–6, 10]. However, unlike previous studies [4–6, 10], leading children to believe that other children had eaten no food (low intake norm) did not influence children to reduce their intake relative to the no-norm and control condition. A possible explanation may be that a floor effect was produced, whereby the low intake observed in the no norm and control conditions resulted in it not being possible to reduce the carrot intake of children any further.

One important consideration in this study is social context. We exposed children to information about the eating behaviour of previous children in a very specific social context, i.e., other children ate like this in this study. Adults appear to be influenced by social norm messages about the healthy eating habits of others [9], so future studies could investigate the effectiveness of less context specific social norm messages for increasing fruit and vegetable intake in children. In addition, the current study investigated carrot intake, therefore, it is not possible to generalise these results to the consumption of other, less common or liked vegetables. Moreover, our sample was predominantly normal-weight, therefore, it is not possible to make conclusions about the behaviour of overweight children.

## Additional file

Additional file 1:
**Supplemental material.** (DOCX 15 kb)
