# Supplementary material for: Perceived eating norms and vegetable consumption in children
Source: Int J Behav Nutr Phys Act. 2015 Oct 14;12:135. doi: 10.1186/s12966-015-0296-z (PMC4604721; doi:10.1186/s12966-015-0296-z)
Supplement: Additional file 1: — Supplemental material. (DOCX 15 kb) [file 12966_2015_296_MOESM1_ESM.docx]

Supplemental material

In order to examine whether BMI (*z-*score), age, gender, carrot liking, or usual fruit and vegetable consumption moderated the effect of condition on the amount of carrots eaten, we conducted a series of further analyses. We found no evidence that any of these variables moderated the effect of condition on amount of carrots eaten.

*BMI (z-score)*

A 4 (condition) x 2 (BMI: normal-weight, overweight) ANOVA was conducted. There was no significant main effect of BMI (*z*-score) on the amount of carrots eaten [F(2, 132)=.98, p=.38, ƞp^2^ =.02], and no significant interaction between condition and BMI on the amount of carrots eaten [F(5,132)=.43, p=.83, ƞp^2^ =.02].

*Age (year group)*

A 4 (condition) x 5 (year group: years 2, 3, 4, 5, 6) ANOVA was conducted. There was no significant main effect of age on the amount of carrots eaten [F(4,123)=.65, p=.63, ƞp^2^ =.02], and no significant interaction between condition and age on the amount of carrots eaten [F(12,123)=.34, p=.98, ƞp^2^ =.03].

*Gender*

A 4 (condition) x 2 (gender) ANOVA was conducted. There was no significant main effect of gender on the amount of carrots eaten [F(1, 135)=.049, p=.83, ƞp^2^ <.001] and, there was no significant interaction between condition and gender on the amount of carrots eaten [F(3, 135)=.18, p=.91, ƞp^2^ = .004].

*Carrot Liking*

A 4 (condition) x 2 (carrot liking: like, dislike) ANOVA was conducted. There was a significant main effect of carrot liking on the amount of carrots eaten [F(1,135)=21.43, p<.001, ƞp^2^ =.14], whereby, children who liked carrots ate significantly more carrots than children who did not like carrots. However, there was no significant interaction between condition and carrot liking on the amount of carrots eaten [F(3,135)=.45, p=.72, ƞp^2^=.01].

*Usual fruit and vegetable consumption*

A 4 (condition) x 2 (usual intake: high, low) ANOVA was conducted. There was no significant main effect of usual fruit and vegetable consumption on the amount of carrots eaten [F(1,135)=.05, p=.82, ƞp^2^ <.001], and there was no significant interaction between condition and usual fruit and vegetable consumption on the amount of carrots eaten [F(3,135)=.41, p=.74, ƞp^2^ =.009].
